# Considerations for prostheses choice in multiple valve surgery

**DOI:** 10.1186/s13019-021-01631-7

**Published:** 2021-09-16

**Authors:** Ahmad Poostizadeh, W. R. Eric Jamieson, A. Ian Munro, Robert T. Miyagishima, Hilton Ling, Guy J. Fradet, Michael T. Janusz, Lawrence H. Burr

**Affiliations:** Department of Surgery, Vancouver Coastal Health Research Institute, University of British Columbia, 2635 (6TH Floor) 2635 Laurel St., Vancouver, BC V5Z1M9 Canada

**Keywords:** Mechanical prostheses, Bioprostheses

## Abstract

**Objectives:**

The prosthesis type for multiple valve surgery (replacement of two or more diseased native or prosthetic valves, replacement of two diseased valves with repair/reconstruction of a third, or replacement of a single diseased valve with repair/reconstruction of a second valve) remains inadequately evaluated. The clinical performance of multiple valve surgery with bioprostheses (BP) and mechanical prostheses (MP) was assessed to compare patient survival and composites of valve-related complications.

**Methods:**

Between 1975 and 2000, 1245 patients had multiple valve surgery (BP 785, mean age 62.0 ± 14.7 years; and MP 460, mean age 56.9 ± 12.9 years). There were 1712 procedures performed [BP 969(56.6%) and MP 743(43.4%). Concomitant coronary artery bypass (conCABG) was BP 206(21.3%) and MP 105(14.1%) (p = 0.0002). The cumulative follow-up was BP 5131 years and MP 3364 years. Independent predictors were determined for mortality, valve-related complications and composites of complications.

**Results:**

Unadjusted patient survival at 12 years was BP 52.1 ± 2.1% and MP 54.8 ± 4.6% (p = 0.1127), while the age adjusted survival was BP 48.7 ± 2.3% and MP 54.4 ± 5.0%. The predictors of overall mortality were age [Hazard Ratio (HR) 1.051, p < 0.0001], previous valve (HR 1.366, p = 0.028) and conCABG (HR 1.27, p = 0.021). The actual freedom from valve-related mortality at 12 years was BP 85.6 ± 1.6% and MP 91.0 ± 1.6% (actuarial p = 0.0167). The predictors of valve-related mortality were valve type (BP > MP) (2.61, p = 0.001), age (HR 1.032, p = 0.0005) and previous valve (HR 12.61, p < 0.0001). The actual freedom from valve-related reoperation at 12 years was BP 60.8 ± 1.9% and MP85.6 ± 2.1% (actuarial p < 0.001). The predictors of valve-related reoperation were valve type (MP > BP) (HR 0.32, p < 0.0001), age (HR 0.99, p = 0.0001) and previous valve (HR 1.38, p = 0.008)

**Conclusions:**

Overall survival (age adjusted) is differentiated by valve type over 10 and 12 years and valve-related mortality and valve-related reoperation favours the use of mechanical prostheses, overall for multiple valve surgery.

**Supplementary Information:**

The online version contains supplementary material available at 10.1186/s13019-021-01631-7.

## Introduction

The clinical performance of bioprostheses and mechanical prostheses in multiple valve surgery, in the long-term is not clearly delineated. The choice of the type of prosthesis, either mechanical or bioprosthesis, depends on the clinical status of the patient, the confidence of the surgeon in the prosthesis, and the risk of valve-related complications [1].

There have been no randomized trials for multiple valve replacements, the Veterans Administration and Edinburgh trials evaluated only aortic and mitral valve prostheses^.^ [2, 3]. Kassai and colleagues [4] have reported a meta-analysis of the randomized trials comparing bioprostheses and mechanical prostheses. Retrospective studies have predominantly provided support for double valve replacement [aortic valve replacement and mitral valve replacement (MVR)] over aortic valve replacement (AVR) and mitral valve repair (MVr) and the reverse alternative opinion, AVR and MVr over double valve replacement [5–9] The major compelling evidence is for double valve replacement with mechanical prostheses, especially in mitral valve rheumatic disease.

Other retrospective studies have been relatively few and some of limited follow-up [10–18]. At 10 years, bioprostheses in multiple valve replacement provide less valve-related morbidity but equivalent freedom from reoperative mortality of bioprostheses and mechanical prostheses [11]. Also at 10 years, mechanical prostheses have been documented to provide better long-term results for triple valve replacement than bioprostheses [13].

Additional evidence for reoperation and major thromboembolism with and without hemorrhage provided no evidence for either bioprostheses or mechanical prostheses in multiple valve replacement [12]. Multiple valve replacement has been identified to result in higher reoperative mortality than reoperation for isolated aortic and mitral valve replacement [14].

The purpose of the study was to determine the clinical performance of multiple valve surgery (replacement of two or more diseased native or prosthetic valves, replacement of two diseased valves with repair of a third or replacement of a single diseased valve with repair/reconstruction of a second valve) with bioprostheses and mechanical prostheses by assessment of patient survival and composites of valve-related complications.

## Materials and methods

This is a retrospective study of prospectively collected data from the University of British Columbia Cardiac Valve Database. The database received annual renewal from the University of British Columbia Research Ethics Board, with a formal consenting process of patients. It included all patients who had multiple valve surgery at the University of British Columbia between 1975 and 2000. For the study multiple valve surgery incorporated patients who had multiple valve procedures as the initial procedure or previous single valve replacement, whether or not the previous prosthesis was replaced or not and replacement at another position.

The 1996 and 2008 publications, “Guidelines for Reporting Morbidity and Mortality After Cardiac Valvular Operations” and “Guidelines for Reporting Morbidity and Mortality After Cardiac Valve Interventions” were used for definitions of valve-related complications, categorization, and statistical methods in this study [19, 20]. Valve-related reoperation and mortality were as defined in these guidelines. Valve-related morbidity was defined as permanent neurological or functional impairment (deviation from the previous guidelines) [21, 22]. Valve-related reoperation and mortality included structural valve deterioration (SVD), non-structural dysfunction (NSD) [inclusive of periprosthetic leak (PPL)], prosthetic valve endocarditis (PVE), and thromboembolism and antithrombotic hemorrhage (TE + ATH). Valve-related mortality also included sudden unexplained deaths (SUD). Patient survival and freedom from valve-related complications were determined by Kaplan–Meier actuarial analysis and expressed as percentage of patients ± standard error. Early mortality was included in the survival analysis. Differences were assessed by log-rank statistic. Freedom from valve-related complications and composites of complications were also evaluated by cumulative incidence or actual methodology using modified Kaplan–Meier analysis [21, 22]. Comparison of continuous and categorical variables was carried out using standard methodology. Evaluation of performance of prostheses by linearized occurrence rates (%/patient-year) had the linearized occurrence rates tested by log likelihood ratio statistics.

The analysis of valve-related mortality and valve-related reoperation is based on procedure, a logic extensively utilized by the authors. Valve-related mortality occurred either at the time of classification as multiple valve surgery or later as a repeat reoperation mortality or a non-operative valve-related complication. The freedom of valve-related reoperation incorporated the first reoperation whether occurring as a previous operation, multiple valve surgery procedure having had a previous procedure, or a subsequent procedure following the primary multiple valve surgery procedure.

The predictors of performance were determined for multiple valve surgery by univariate and multivariate Cox proportional hazard regression analysis. The variables considered were: valve type, age, gender, previous coronary artery bypass, previous valve procedure, concomitant coronary artery bypass, renal disease, diabetes mellitus, left ventricular dysfunction (EF—Ejection Fraction), chronic obstructive pulmonary disease (COPD) and rhythm. Individual factors potentially predictive of overall mortality and freedom from valve-related complications and composites of complications were initially analyzed using univariate analysis. To determine independent predictors of mortality and valve-related complications, all variables with a *p *value < 0.10 by univariate analysis were entered as covariates in the multivariate analysis by Cox proportional hazard stepwise logistic regression. A value of *p* < 0.05 was considered significant and HR designated hazard ratio.

The distribution of patients and the procedures performed is documented in Table 1. The patient population of 1245 patients consisted of 565 males and 680 females. Of the total population, 785 had bioprostheses (mean age 62.0 ± 14.7 years) and 460 had mechanical prostheses (mean age 56.9 ± 12.9 years) (p < 0.0001). These 1245 patients had 1712 operative procedures, inclusive of a previous procedure prior to being classified as multiple valve surgery, or subsequent procedure(s) after being classified as multiple valve surgery.

**Table 1:** Distribution of patients and procedures

	Total	BP (%)	MP (%)	p value
Patients	1245	785 (63.0)	460 (36.0)	
Gender (females)	680	447 (56.9)	233 (50.6)	**0.036**
Procedures	1712	969 (56.6)	743 (43.4)	
Previous procedures	298 (17.4)	241 (24.9)	57 (7.7)	**< 0.001**
Post multiple surgery procedures	169 (9.9)	53 (5.5)	116 (15.6)	**< 0.001**

Bold indicates significant p values

The 1245 patients had 976 patients considered as the initial multiple valve procedures and 269 patients with previous single valve procedures (228 two procedures, 37 three procedures and 4 four procedures). The recorded pre-operative rhythm was atrial fibrillation or paced in 17.1% of bioprostheses patients and 38.7% of mechanical prostheses patients.

The 1712 operative procedures in the 1245 patients were considered the 1245 procedures when classification as multiple surgery and 467 procedures classified as previous or subsequent procedures. The 467 procedures were determined as 380 second procedure, 74 third, 11 fourth and 2 fifth procedure. This population also did previously have 81 repair/commissurotomy procedures (BP–38 and MP–43).

Of the patients with previous procedures there were 241 BP replacements and 57 MP replacements (Table 1) prior to the patients qualifying as multiple valve surgery (BP > MP, p < 0.001). Following qualifying as multiple surgery 169 subsequent procedures were performed, 53 BP replacements and 116 MP replacements (MP > BP, p < 0.001) (Table 1).

The total multiple surgery experience of the study centres also included 74 patients with 77 procedures in a mixed BP- MP combination. This group of mixed prostheses population were not considered in the analysis.

The procedures performed in the BP and MP groups are complex. The majority of the procedures were double valve replacement (540BP and 501MP) and only 12BP and 5MP having triple valve replacement. Of 70BP double valve replacements and 82MP double valve replacements, tricuspid valve repair (TVr) was incorporated. A minority (62 procedures) of single valve replacements had repairs of an alternate valve (AVr, MVr and TVr) and are considered as multiple valve surgery in the analysis. One double valve replacement had repair of a third valve. Two single valve replacements had repair of two valves. The total population is classified as multiple valve surgery (MVS) not just double or triple valve replacement.

Of the total bioprostheses implanted, 86.4% were porcine bioprostheses, 13.3% were pericardial bioprostheses and 0.3% were homografts. Of the total mechanical prostheses implanted, 98.4% were bileaflet, 1.0% were monoleaflet and 0.6% were ball-cage prostheses. These incorporated all bioprostheses and mechanical prostheses in all positions.

The clinical characteristics of the procedures performed incorporated previous coronary artery bypass (CABG) in 3.4% (33) of BP and 3.3% (25) of MP (pNS). Concomitant CABG was performed in 21.3% (206) of BP and 14.1% (105) of MP (p = 0.0002).

The total follow-up was 5131 patient-years for BP and 3364 patient-years for MP. The follow-up was 98.2% complete, with a nine-month closing interval in 2004. At the latest follow-up 12.6% of bioprostheses patients were managed on anticoagulation with coumadin and presumably all the patients with mechanical prostheses patients, even though the documentation was incomplete.

## Results

The early mortality, for the multiple valve procedure, for the BP population was 8.4% and for the MP population was 6.5% (p = 0.214). The unadjusted survival was not different, overall, at 12 years, MP 54.8 ± 4.6% and BP 52.1 ± 2.1(p = 0.1127) (Additional file 1: Figure E1). The age adjusted survival was different, at 12 years, 54.5 ± 5.0% for MP and 48.7 ± 2.3% for BP. Valve type was not predictive of survival. The predictors were age (HR 1.051, p < 0.0001), previous valve procedure (HR 1.366, p = 0.028) and conCABG (HR 1.276, p = 0.021). Valve type was not predictive of overall mortality or of mortality in any of the specific age groups.

**Fig. 1:**
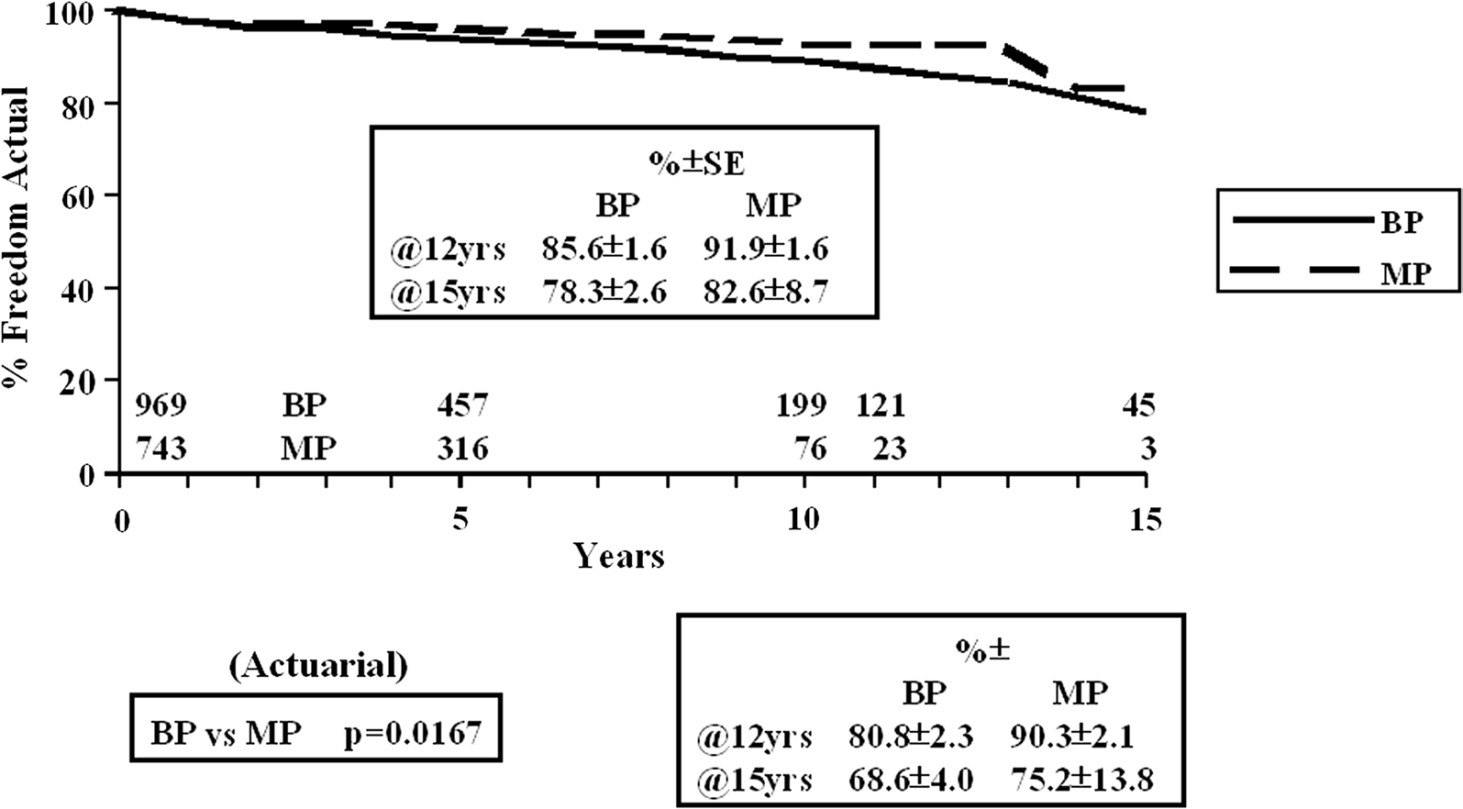
Freedom from valve-related mortality (actual and actuarial) (BP vs. MP)

The assessment of predictors of each of the valve-related complications revealed valve-type was predictive of antithrombotic hemorrhage (HR 1.80, p = 0.037; MP > BP), prosthetic valve endocarditis (HR 1.79, p = 0.041; BP > MP) and structural valve deterioration (HR 71.85, p < 0.0001; BP > MP).

There were 501 deaths overall, BP—337 and MP—164. The overall differentiation for overall mortality was BP6.6%/pt.-year and MP4.9%/pt.-year (p = 0.001). There were 133 valve-related deaths (BP-100 and MP-33). The cardiac deaths totalled 199 (BP-132 and MP-67). The non-cardiac deaths were 169 (BP-105 and MP-64). Of the BP overall mortality 29.7% were valve-related and 31.2% non-cardiac; of MP 20.1% were valve-related and 39.0% non-cardiac. The cardiac deaths were similar—BP39.2% and MP40.9%. The prosthesis-type was differentiated only for valve-related mortality—BP1.95%/pt.-year and MP0.98%/pt.-year (p = 0.0003).

The detailed etiology of valve-related mortality and valve-related reoperation is presented in Tables 2 and 3. Valve-related mortality occurred in 100BP with 11 sudden, unexplained deaths and 33MP with 4 sudden unexplained deaths (Table 2). There were 42 BP deaths due to structural valve deterioration (SVD).

**Table 2:** Etiology of valve-related mortality

(Events, %/%/Pt-Year)						
	Total	BP		MP		p value
ATH	17 (12.8%)	8 (8.0%)	0.16	9 (27.3%)	0.27	0.270
NSD	15 (11.3%)	7 (7.0%)	0.14	8 (24.2%)	0.24	0.283
PVE	21 (15.8%)	15 (15.0%)	0.29	6 (18.2%)	0.18	0.292
SVD	42 (31.6%)	42 (42.0%)	0.82	0 (0.0%)	0.0	**< 0.0001**
TE	20 (15.0%)	16 (16.0%)	0.31	4 (12.1%)	0.12	0.0603
TB	3 (2.3%)	1 (1.0%)	0.02	2 (6.1%)	0.06	0.3442
SUD	15 (11.3%)	11 (11.0%)	0.21	4 (12.1%)	0.12	0.2931
Total	**133 (100.0%)**	**100 (100.0%)**	**1.95**	**33 (100.0%)**	**0.98**	**0.0003**

Bold indicates significant p values

**Table 3:** Etiology of valve-related reoperation

(Events, %, %/%/Pt-Year)						
	Total	BP		MP		p value
NSD	104 (25.6%)	59 (17.3%)	1.1	45 (69.3%)	1.3	0.4463
PVE	42 (10.3%)	29 (8.5%)	0.57	13 (20.0%)	0.39	0.2444
SVD	254 (62.4%)	254 (74.3%)	5.0	–	0.0	**< 0.0001**
TE	1 (0.2%)	0 (0.0%)	–	1 (1.5%)	0.03	0.1734
TB	6 (1.6%)	0 (0.0%)	–	6 (9.2%)	0.18	**0.0009**
Total	**407 (100.0%)**	**342 (100.0%)**	**6.67**	**65 (100.0%)**	**1.93**	**< 0.0000**

Bold indicates significant p values

Valve-related reoperation was performed in 342 BP and 65 MP (Table 3). Of the BP reoperations 254 were for SVD [93 AVR, 108 MVR, 3 TVR and 50 multiple reoperations (49 AVR, 50 MVR and 1 TVR)]. Of the 65 MP reoperations 45 were for periprosthetic leak, and 13 for prosthetic valve endocarditis and 6 for prosthesis thrombosis. The valve-related reoperation rate for BP was 6.67%/pt.-year and for MP was 1.93%/pt.-year (p < 0.000). Early mortality for BP reoperations was 13.5% (46 of 342) and for MP was 16.9% (11 of 65) (p = 0.586).

The freedom from valve-related mortality (actual and actuarial) is detailed in Fig. 1. The actual freedom from valve-related mortality, at 12 years, was BP 85.6 ± 1.6% and MP 91.9 ± 1.6% (actuarial BP 80.8 ± 2.3% and MP 90.3 ± 2.1%, p = 0.0167). Valve type (BP > MP) was an independent predictor of valve-related mortality (HR 2.61, p = 0.001). Age (HR 1.032, p = 0.0005) and previous valve (HR 12.61, p < 0.0001) were also predictive.

The actual freedom from valve-related morbidity at 12 years was BP 99.3 ± 0.3% and MP 99.4 ± 0.4% (actuarial BP 99.1 ± 0.4% and MP 99.3 ± 0.4%, p = 0.7808). There were no predictors of valve-related morbidity (permanent neurologic or functional impairment) identifying valve type or other risk factors.

**Fig. 2:**
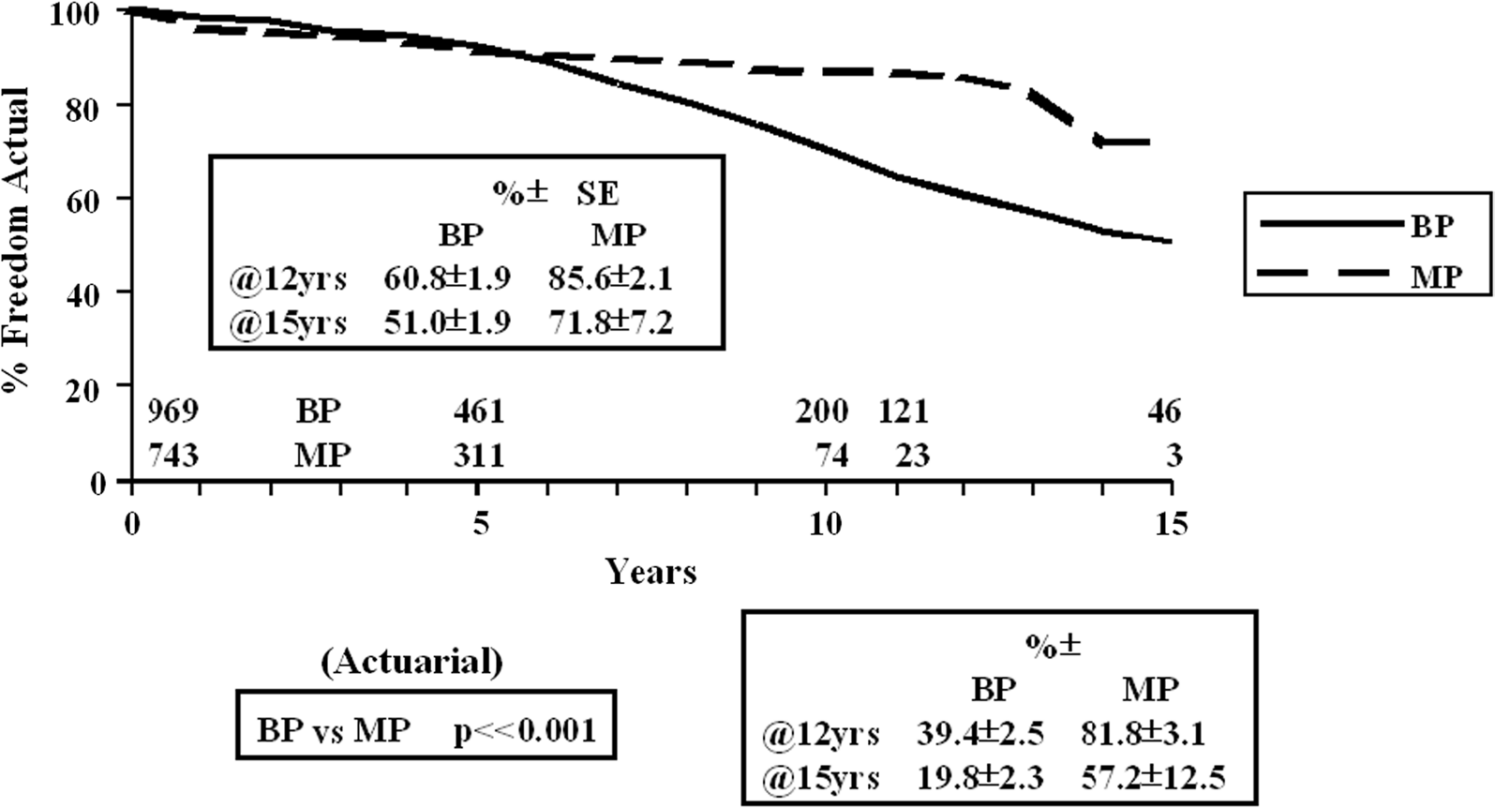
Freedom from valve-related reoperation (actual and actuarial) (BP vs. MP)

The freedom from valve-related reoperation (actual and actuarial) is detailed in Fig. 2. The actual freedom at 12 years was BP 60.8 ± 1.9% and MP 85.6 ± 1.7% (actuarial BP 39.4 ± 2.5% and MP 81.8 ± 3.1%, p < 0.001). The independent predictors of valve-related reoperation were valve-type (HR 0.32, p < 0.0001, MP > BP); age (HR 0.99, p = 0.0001) and previous valve (HR 1.38, p = 0.008).

The freedom from valve-related reoperation for bioprostheses and mechanical prostheses by age groups (≤ 50, 51–60, 61–70, > 70 years) by actual and actuarial methodology was evaluated. All age groups support the use of mechanical prostheses over bioprostheses. For the age group 61–70 years, the actual freedom, at 12 years, was MP 91.1 ± 2.5% and BP 64.7 ± 3.8% (actuarial MP 88.5 ± 3.3% and BP 42.0 ± 5.7%). For the > 70 years age group, the actual freedom, at 12 years, was MP 96.9 ± 2.1% and BP 89.6 ± 2.6% (actuarial MP 96.4 ± 2.5% and BP 77.5 ± 7.0%).

The freedom from combined composites of valve-related complications (reoperation, mortality and morbidity) at 12 years, was 32.9 ± 2.3% for BP and 73.6 ± 3.3% for MP, actuarial (p < 0.0001) (Additional file 1: Table E3 and Additional file 1: Figure E2).

## Discussion

There is limited documentation on the performance of prostheses type for multiple valve surgery [11–16]. This study is the first to report 12-year results of surgery comparing the performance of bioprostheses and mechanical prostheses for multiple valve surgery. All the reports have been retrospective, non-randomized studies, including this current study [11–16]. The question posed is whether patients who have the need for primary surgery and may need reoperative surgery for other valve disease should have mechanical prostheses or bioprostheses in the context of double or triple valve surgery. The evaluation was considered for generally three options available to the cardiac surgeons, namely, double valve replacement with mechanical prostheses, double valve replacement with bioprostheses, and aortic valve replacement combined with mitral valve repair. The procedures performed were primarily double valve and triple valve replacements. The double valve pathology and consequences on ventricular function and the tricuspid valve necessitated tricuspid valve reconstruction in 11.3% (193) of the procedures. Single valve replacements with repair/reconstruction of another valve was performed in only 2.8% of the patients. Previous valve replacement procedures, prior to being considered or having multiple valve surgery, had been performed in 17.4% (298) of the procedures. Following having been classified as multiple valve surgery 169 or 9.9% of patients had subsequent valve procedures.

This study does not differentiate unadjusted survival between the prosthesis types at 12 years, 52.% for bioprostheses and 55% for mechanical prostheses, even though the bioprosthesis population had a mean age difference of 5.1 years over the mechanical prosthesis population. The age adjusted survival, although was 49% for bioprostheses and 54% for mechanical prostheses. The significant differentiations incorporate valve-related mortality and valve-related reoperation. The freedom from valve-related mortality was, at 12 years, actual 86% for bioprostheses and 92% for mechanical prostheses while actuarial 81% and 90%, respectively. This is not a major differentiation with the hazard ratio being 2.6 for bioprostheses over mechanical prostheses while previous valve surgery had a hazard ratio of 12.6.

The major differentiating feature was freedom from valve-related reoperation, actual 61% for bioprostheses and 86% for mechanical prostheses at 12 years. The significant actuarial freedom, at 12 years, was 39% for bioprostheses and 82% for mechanical prostheses. Mechanical valve type was protective from reoperation, while previous valve surgery was predictive. This data definitely provides support for mechanical prostheses for multiple valve surgery. Valve-related mortality did differentiate the prosthesis type but influenced predominately by prior single valve replacement, which converted to multiple valve surgery at the time of reoperative procedure at a different site.

There have been two additional reports of multiple valve replacement from this study’s centre; in 1995 and 2000. In 1995, Munro and colleagues [11] reported 10-year results on 494 patients with 533 operations between 1975 and 1992. The freedom from valve-related mortality, at 10 years, was not different, by prostheses type. The freedom from valve-related reoperation also did not differentiate the groups. The overall reoperative early mortality was 16%. In 2000, Jamieson and co-investigators [12] could not differentiate prosthesis type for multiple valve replacement based on predictors of performance whereas predictors supported bioprostheses for aortic valve replacement and mechanical prostheses for mitral valve replacement. These studies were conducted with limited extent of long-term evaluation.

The report by Caus and colleagues [15] identified the risk of reoperation for structural valve deterioration of bioprostheses is acceptable for single reoperative valve replacement as opposed to multiple reoperative valve replacement. The authors, in a later publication, compared bioprostheses and mechanical prostheses in aortic valve replacement and mitral valve replacement identifying the risk of reoperative mortality was equivalent to lethal hemorrhage [16].

Only three other reports address prosthesis-type in multiple valve replacement. Brown and colleagues [13] provided support for mechanical prostheses in triple valve replacement by documenting the reduced need for reoperation and accompanying complications. Christenson and co-authors [14] identified a higher mortality for multiple valve replacement reoperations than valve reoperations for single valve replacement surgery. The comparative results of bioprostheses and mechanical prostheses in mitral and aortic valve replacements have been reported from the authors’ centre [21, 22].

Carrier and colleagues [23] reported mid-term results that survival with bioprostheses and mechanical prostheses was acceptable in triple valve surgery, and that aortic valve replacement with mechanical prostheses and mitral valve repair and tricuspid valve repair should be a considered alternative. The results of triple valve surgery were reported by Alsoufi and co-investigators [24], in 2006, revealing in 174 patients who had surgery between 1990–2004 that operative mortality was 13%. These results are not different from the authors study extending over a longer time frame to 1975. The study did not address mechanical or bioprosthetic valve results.

In the study only 17 or 1.3% of patients had aortic valve replacement and mitral valve repair, identifying the unpopularity of mitral valve repair/reconstruction in multiple valve surgery within our centre. There have been considerable publications addressing this management modality. Szentpetry and co-authors [7], in 1997, commenced assessment of aortic valve replacement and mitral valve repair rather than mitral valve replacement. They recommended, with evaluation of 38 patients, that with low surgical mortality and reduced morbidity mitral repair should be considered an alternative to replacement. Gillinov and investigator [8] reported, in 2003, on an extensive experience with surgery for double valve disease (mitral valve replacement—518 and mitral valve repair—295). This study reported improved survival benefit with mitral valve repair including elderly, coronary artery disease, left ventricular dysfunction, rheumatic and non-rheumatic disease. The durability of mitral valve repair even with rheumatic disease exceeds that of bioprostheses in the mitral valve position. The preferred strategy, from the attempted bias controlled study, was that the mitral valve amenable to repair in double valve disease should be repaired rather than replaced. Ho and investigators [10], in 2004, also recommended that mitral valve repair should be attempted if repair suitable and double valve replacement reserved for complex mitral valve disease. Talwar and colleagues [9], provided a report in 2007, demonstrating that aortic valve replacement with mitral repair provides better event-free survival than double valve replacement without a better actuarial survival advantage. Reoperation rates, although, were higher in the mitral repair group, but thromboembolic rates were lower thus resulting in a better event-free survival in the mitral repair and aortic valve replacement group.

The alternative opinion was proposed by Hamamoto and Kuwaki and co-investigators [5, 6]. Hamamoto reported on 379 patients (double valve replacement 299 and aortic valve replacement with mitral valve repair 80) with the freedom from reoperation at 15 years being 54% and 15%, respectively. The causes of reoperation were progression of mitral pathology and structural valve deterioration of a bioprosthesis. The freedom from mitral valve repair reoperation at 15 years was 63% for non-rheumatic disease and only 5% for rheumatic disease. The mortality from reoperation was similar. The study recommended mechanical prostheses for double valve replacement as the procedure of choice and that mitral valve repair should not be used in mitral valve rheumatic disease.

There are limitations in this study. The complexity of multiple valve surgery is evident but cannot be avoided if all patients are considered as in this study. To address only double valve replacement, the majority would have been aortic and mitral valve replacement but double valve replacement occurs when tricuspid valve replacement and pulmonary valve replacement is combined with a prosthesis replacement in another site. The complexity of the population is also contributed to by previous single valve replacements and subsequent valvular surgery due to valve-related complications or cardiomyopathy. The patient population does not afford the consideration of mitral valve repair or replacement with aortic valve replacement in double valve surgery. Also we have not evaluated tricuspid valve repair or replacement in triple valve surgery because of low number of tricuspid valve replacements in triple valve surgery. The advances in myocardial preservation and extra-corporeal circulation over the 25 year period is not addressed in the study and must be considered a limitation of the study. Atrial fibrillation surgery was not incorporated in operative surgery until after 2000. The limited follow-up between 12 and 15 years with the mechanical prosthesis population is a limitation of the study but, in our opinion, does not influence the recommendations of the study.

The study provided extensive documentation in support of the overall recommendation that mechanical prostheses are recommended for multiple valve surgery. Valve type was not a predictor of survival, only previous prosthesis and concomitant CABG. The overall mortality for bioprostheses was predominant for valve-related mortality (29.7% vs. 20.1%) and mechanical prostheses for non-cardiac mortality (39.0% vs. 31.2%). Valve-related mortality was influenced by valve type and by previous valve. The actual freedom (cumulative analysis), at 12 years, from valve-related mortality was 92% for mechanical prostheses and 86% for bioprostheses The linearized rate of valve-related mortality was 1.95%/pt-year for bioprostheses and 0.9%pt-year for mechanical prostheses.

The actual freedom (cumulative analysis), at 12 years, from valve-related reoperation was 86% for mechanical prostheses and 61% for bioprostheses. The predominant predictor of valve-related reoperation was previous prosthesis while protective by advancing age and mechanical prostheses. The linearized rate of bioprostheses structural valve deterioration was 5.0%/pt-year. Structural valve deterioration was predictive by valve type with a bioprosthesis hazard ratio of 72. Previous valve replacement was 6 times more common in the bioprostheses group and post-reoperation 4.7 times more common. The early mortality from valve-related reoperation was 13.5% for bioprostheses and 16.9% for mechanical prostheses. The linearized rate of valve-related reoperation was 6.7%/pt-year for bioprostheses and 1.9%/pt-year for mechanical prostheses. Mechanical prostheses did influence hemorrhage over bioprostheses.

The final summary detail is the freedom from composites of all valve-related complications at 12 years, 73.6% for mechanical prostheses and 32.9% for bioprostheses.

The only recent published in 2018 by Leone and colleagues from the report on triple valve surgery was RERIC (Emilia Romagna Cardiac Surgery Registry) investigators [25]. This study did not address the type of prostheses for multiple (triple) valve surgery. The early mortality in the 211 patients was 7.9%, similar to the authors’ long term study, The predictors of in-hospital mortality were renal insufficiency, concomitant coronary artery bypass surgery and previous cardiac surgery.

## Conclusions

The experience in 1245 patients having had 1712 previous, initial or subsequent procedures has determined that the freedom from valve-related reoperation at 12 years provides evidence to support mechanical prostheses, for all age groups (even for patients over 70 years of age) to avoid/reduce valve-related mortality and especially valve-related reoperation, instead of bioprostheses for multiple valve surgery. The higher prevalence of prior surgery in patients with multiple valve disease should discourage the use of bioprosrheses because of the risk of subsequent reoperative surgery with the establishment of multiple valve surgery. The overall recommendation for multiple valve surgery is mechanical prostheses.

## Supplementary Information

**Additional file 1.Figure E1.** Patient survival—bioprostheses and mechanical prostheses populations. **Figure E2.** Freedom from the composites of valve-related reoperation, valve-related residual morbidity and valve-related mortality. **Table E1.** Etiology of overall mortality. **Table E2.** Etiology of early mortality of valve-related reoperation. **Table E3.** Freedom from combined composites of valve-related complications (reoperation, mortality and morbidity) by procedures—bioprostheses (969) and mechanical prostheses (743), for age groups at 10 years.

## Data Availability

The authors had availability of all data over years.

## Abbreviations

BP: Bioprostheses
MP: Mechanical prostheses
CABG: Coronary artery bypass graft
conCABG: Concomitant CABG
HR: Hazard ratio
AVR: Aortic valve replacement
MVR: Mitral valve replacement
TVR: Tricuspid valve replacement
ARr: Aortic valve repair
MVr: Mitral valve repair
TVr: Tricuspid valve repair
SVD: Structural valve deterioration
NSD: Non-structural dysfunction
TE: Thromboembolism
ATH: Antithrombotic hemorrhage
PVE: Prosthetic valve endocarditis
PPL: Peri-prosthetic leak
EF: Ejection fraction
COPD: Chronic obstructive pulmonary disease
SAV: Supra-valvular valve
HP: St Jude Medical High Performance
MVS: Multiple valve surgery
