# Supplementary material for: Considerations for prostheses choice in multiple valve surgery
Source: J Cardiothorac Surg. 2021 Sep 16;16:262. doi: 10.1186/s13019-021-01631-7 (PMC8447611; doi:10.1186/s13019-021-01631-7)
Supplement: Supplementary file 1 — Additional file 1.Figure E1. Patient survival—bioprostheses and mechanical prostheses populations. Figure E2. Freedom from the composites of valve-related reoperation, valve-related residual morbidity and valve-related mortality. Table E1. Etiology of overall mortality. Table E2. Etiology of early mortality of valve-related reoperation. Table E3. Freedom from combined composites of valve-related complications (reoperation, mortality and morbidity) by procedures—bioprostheses (969) and mechanical prostheses (743), for age groups at 10 years. [file 13019_2021_1631_MOESM1_ESM.doc]

**Table E1 – Etiology of Overall Mortality**

|  | | | | | | | | | |
| --- | --- | --- | --- | --- | --- | --- | --- | --- | --- |
|  | **Total** | | **BP** | | | **MP** | | | **p-Value** |
|  |  |  | **No.** | **%** | **%/Pt-Yr** | **No.** | **%** | **%/Pt-Yr** |  |
| **Valve -Related** | 133 | 26.5 % | 100 | 29.7 % | 1.95 | 33 | 20.1 % | 0.98 | **0.0003** |
| **Cardiac** | 199 | 39.7 % | 132 | 39.2 % | 2.57 | 67 | 40.9 % | 1.99 | 0.0840 |
| **Non -Cardiac** | 169 | 33.7 % | 105 | 31.2 % | 2.05 | 64 | 39.0 % | 1.9 | 0.6447 |
|  | | | | | | | | | |
| **Total** | 501 | 100 % | 337 | 100 % | 6.57 | 164 | 100 % | 4.88 | **0.0015** |

**Table E2 – Etiology of Early Mortality of Valve-Related Reoperation**

|  | | | | |
| --- | --- | --- | --- | --- |
|  | **Total** | **BP** | **MP** | **p-Value** |
| **NSD** | 11 (10.6 %) | 5 (8.5 %) | 6 (13.3 %) | 0.525 |
| **PVE** | 9 (21.4 %) | 5 (17.2 %) | 4 (30.8 %) | 0.422 |
| **SVD** | 36 (14.2 %) | 36 (14.2 %) | — | **<0.0001** |
| **TB** | 1 (16.7 %) | — | 1 (16.7 %) | **<0.0001** |
| **Total** | **57 (14.0 %)** | **46 (13.5 %)** | **11 (16.9 %)** | 0.586 |
|  | | | | |

**Table E3 – Freedom from Combined Composites of Valve-Related Complications (Reoperation, Mortality and Morbidity) by Procedures – Bioprostheses (969) and Mechanical Prostheses (743), for Age Groups at 10 Years**

| Age Groups | BP | | MP | | p-Values |
| --- | --- | --- | --- | --- | --- |
| (Years) | N |  | n |  |  |
| **<51** | **190** | **38.3±3.9 (58)** | **222** | **74.3±4.3 (30)** | **<0.0001** |
| 51-60 | 191 | 58.5±4.0 (85) | 179 | 74.7±5.0 (18) | 0.105 |
| **61-70** | **250** | **45.1±4.8 (39)** | **249** | **76.0±5.5 (18)** | **0.0001** |
| ≥70 | 338 | 66.9±5.4 (14) | 93 | 81.5±7.3 (5) | 0.075 |

**Figure E1 Patient Survival - Bioprostheses and Mechanical Prostheses Populations**


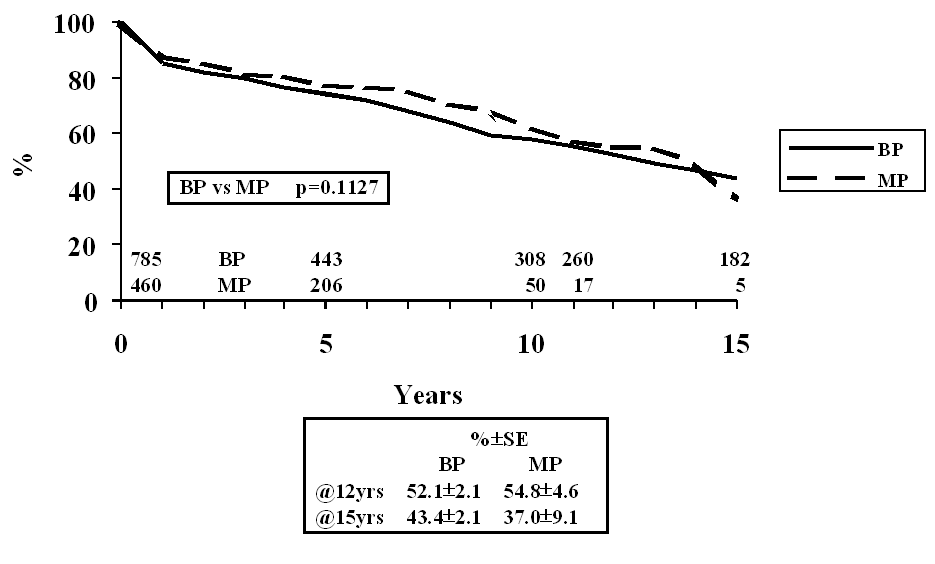


**Figure E2 Freedom from the Composites of Valve-Related Reoperation, Valve-Related Residual Morbidity and Valve-Related Mortality**
